# Anthocyanin Structural Types and Stability: Implications for Natural Colorant Applications in Food Systems

**DOI:** 10.3390/foods15122080

**Published:** 2026-06-08

**Authors:** Munir Ahmed, Hua Zhang, Yun Xiong

**Affiliations:** 1 Department of Food Nutrition and Safety, College of Pharmacy, Jiangxi University of Chinese Medicine, Nanchang 330004, China; munirahmed@jxutcm.edu.cn; 2 Key Laboratory of Modern Preparation of Chinese Medicine, Ministry of Education, Department of Development and Evaluation of Dietary Functional Products, Jiangxi University of Chinese Medicine, Nanchang 330004, China

**Keywords:** anthocyanins, 3-deoxyanthocyanidins, structure–property relationship, stability, food matrices

## Abstract

Anthocyanins are promising natural food colorants because of their vibrant hues and health benefits, which have prompted recent research efforts. Although various techniques have been developed to externally stabilize anthocyanins and preserve their color and stability during food processing and storage, the impact of their molecular structure on stability within food matrices remains insufficiently explored. This review summarizes the stability of anthocyanins in relation to their structural diversity, transformations, and environmental factors. Features such as hydroxylation, methoxylation, glycosylation, acylation, and related structures like 3-deoxyanthocyanidins, pyranoanthocyanins, and polymeric pigments are examined concerning hydration, oxidation, and chromophore stability. The mechanisms involved include electronic effects, steric hindrance, molecular rigidity, co-pigmentation, and interactions with the food matrix. The color of anthocyanins is highly pH-dependent, transitioning from red in acidic conditions to blue or colorless at neutral and alkaline pH, reflecting underlying structural transformations of the chromophore. Food-processing conditions, including pH changes, heat treatment, light exposure, and interactions with complex matrices, have a significant influence on the stability behavior of these compounds. The structure–property relationships are outlined to guide the use of different anthocyanins in diverse food systems: acidic beverages, neutral products, heat-processed foods, and novel applications. This review provides a fundamental understanding of anthocyanin stability and establishes a scientific basis for utilizing various anthocyanins in current food applications.

## 1. Introduction

Anthocyanins are a class of naturally occurring water-soluble pigments found in plants. Recently, there has been a marked increase in interest in anthocyanins within the food and beverage industry due to their function as natural coloring agents and their potential health benefits. With the growing demand for clean-label foods and heightened concerns over artificial colorants’ safety, anthocyanins have become an attractive option in food applications [1,2,3,4].

However, their unstable nature within the environment greatly restricts their practical application, despite exhibiting good color properties and biological attributes. Anthocyanins are very sensitive to parameters like pH levels, heat, and UV rays, which cause changes in the structure of anthocyanins and make them unstable [5,6,7,8]. The loss of color and structural instability leads to the inability of anthocyanin-rich foods to perform their functions effectively. The instability of anthocyanins relates to their dynamic structural balance in an aqueous solution. The peculiarity of anthocyanin behavior lies in the dynamic shift from one form to another, influencing the stability of color chromatic stability [8]. With the rise in pH values, the tendency of stable flavylium ions is to form less stable carbinol, colorless chalcones, and carbinol pseudobase ions [9]. Furthermore, thermal processing leads to the breaking of glycosidic linkages as well as rupture of the pyran ring with the formation of degraded compounds that possess lighter color intensity than parent anthocyanin molecules. Thermal degradation not only diminishes the visual color quality of anthocyanin-containing foods but also reduces their bioactivity, as the degradation products typically exhibit decreased antioxidant capacity and altered bioavailability compared to the parent compounds [10,11].

The basic chromophore of these pigments, the anthocyanidin aglycone, consists of a flavylium cation core with a C6-C3-C6 system (rings A and B) linked via a pyran ring [4,9,12]. In their naturally occurring forms, anthocyanidins are typically glycosylated to yield anthocyanins, in which one or more sugar moieties (e.g., glucose, xylose, rhamnose) are attached to the aglycone, mainly at the C-3 position. Throughout this review, anthocyanins (i.e., the glycosylated forms) are treated as the principal subjects of discussion, while anthocyanidins (aglycones) are referenced specifically where structural distinction is relevant [13,14]. Structural variation is additionally achieved through modifications in substitution, glycosylation, and acylation of organic acids that can be aromatic, such as p-coumaric and ferulic acids, and aliphatic, such as malic and acetic acids [15,16]. Structural characteristics of anthocyanins that govern their specific physical-chemical properties determine color and chemical stability.

Various approaches have been explored to improve anthocyanin stability during processing and storage, like co-pigmentation and encapsulation [17,18,19]. Numerous studies and reviews focus on extraction, bioactivities, general stability, and external stabilization [1,20,21]. However, to date, few reviews are available that specifically address the relation between anthocyanin molecular structure and stability.

This review focuses on exploring the stability of individual anthocyanins in relation to their molecular structures as well as their performance in model systems and in food applications. The purpose is to create a rationale for the insights and selection of anthocyanins and their application as natural colorants in food systems. Anthocyanins suitable for use as natural food colorants are obtained from a wide range of plant sources. Berries (blueberry, blackberry, raspberry, blackcurrant, elderberry), grapes, red cabbage, purple sweet potato, black carrot, red radish, hibiscus, and butterfly pea flower are among the most widely exploited commercial sources, each providing distinct anthocyanin profiles suited to specific food applications [1,3,20].

This review was prepared based on a structured literature search and thematic synthesis. Relevant publications were retrieved from PubMed, Web of Science, ScienceDirect, and Google Scholar, covering publications from 2021 to 2025. In addition, earlier key studies were included where necessary to provide foundational insights. Search keywords included “anthocyanins”, “3-deoxyanthocyanidins”, “pyranoanthocyanins”, “stability”, “color stability”, “degradation”, “structure-stability relationship”, “glycosylation”, “acylation”, “methoxylation”, “co-pigmentation”, “pH”, “temperature”, “light”, “oxidation”, “food systems”, and “food colorants”. Relevant publications were screened by title, abstract, and full-text evaluation. Only peer-reviewed research and review articles focusing on anthocyanin structural chemistry, functional mechanisms, stability, and related applications were included. Non-English papers, conference abstracts without complete experimental data, and studies not directly relevant to the scope of this review were excluded.

## 2. Structural Diversity of Anthocyanin

Anthocyanins are naturally occurring pigments of which more than 700 individual compounds have been isolated or identified. However, these individual anthocyanins originate from a relatively small number of anthocyanidin aglycones that have been found to occur in different forms after undergoing modification [22,23,24]. Naturally, anthocyanins occur as glycosides with one or more sugar molecules covalently linked to the aglycone (anthocyanidin). The sugar moiety is typically located at the C3 position of the C-ring; however, the C5 and C7 positions are known to be replaced with sugar moieties. Such primary structural modifications directly influence the solubility and chemical stability of the compounds [25].

**Figure 1:**
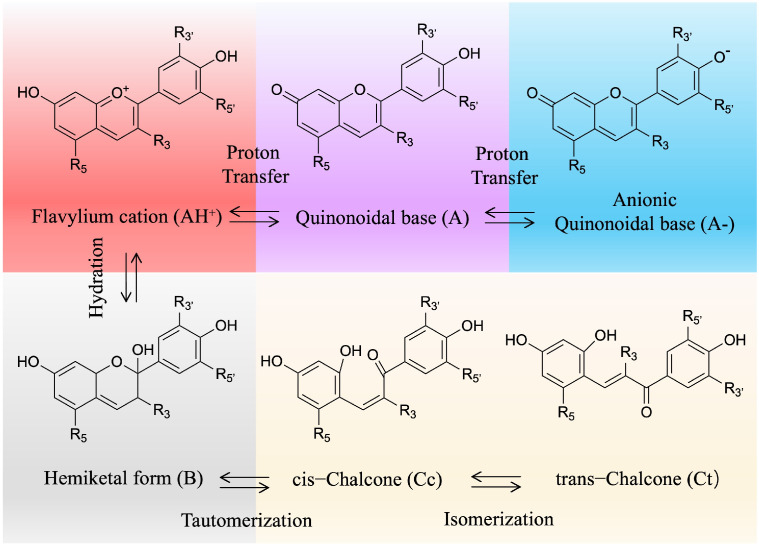
pH−dependent reversible transformation equilibrium of anthocyanin forms. Anthocyanins occur in several interconvertible forms: red flavylium cation (AH^+^, the most stable form), neutral quinoidal base (A), and anionic quinoidal base (A−). These three forms of anthocyanins are in equilibrium with each other via reversible proton transfer reactions. With increasing pH, the flavylium cation (AH^+^) undergoes hydration to the colorless carbinol pseudobase (B), which can further isomerize into cis-chalcone (Cc) and trans−chalcone (Ct).

The color of anthocyanins arises from the chromophoric flavylium cation (AH^+^) with its conjugated π-electron system that absorbs visible light. The color of anthocyanins varies with the reversible equilibrium among different species: flavylium cation (AH^+^), quinoidal base (A or A^−^), colorless carbinol pseudobase (B), and chalcone forms (C or Ct). Correspondingly, the color changes from red and purple to blue to colorless. Such color changes in response to environmental factors make them interesting color systems, based on which numerous responsive materials have been designed [26,27]. The reversible equilibrium of different anthocyanin forms is shown in Figure 1. These equilibria provide a basis for understanding the reversible transformation mechanism and help explain the stability differences in anthocyanin structures.

Variations in anthocyanins mostly stem from modifications to the flavylium cation core, primarily different patterns of substitution of the B-ring, including hydroxy and methoxy groups. These variations affect the molecule’s electronic structures and absorption properties [25]. In addition, glycosylation and subsequent modification of the sugar rings can introduce polarity and solubility into the molecule; further modifications, such as acylation, are often associated with enhanced stability [28]. Moreover, anthocyanins can be divided into more basic subclasses, such as pyranoanthocyanins, which are formed during wine fermentation and aging, as well as other plant tissues, and 3-deoxyanthocyanidins, which lack a hydroxyl group at the C3 position [29,30]. Numerous substitutions, glycosylations, acylations, etc., change the polarity and conformation of anthocyanins, leading to distinctive chemical stability and properties. Many of these compounds have potential applications in food, materials, and biosciences, and it is necessary to clarify in detail the complex structure–property relationships of these natural molecules.

### 2.1. Anthocyanidin Backbones

**Figure 2:**
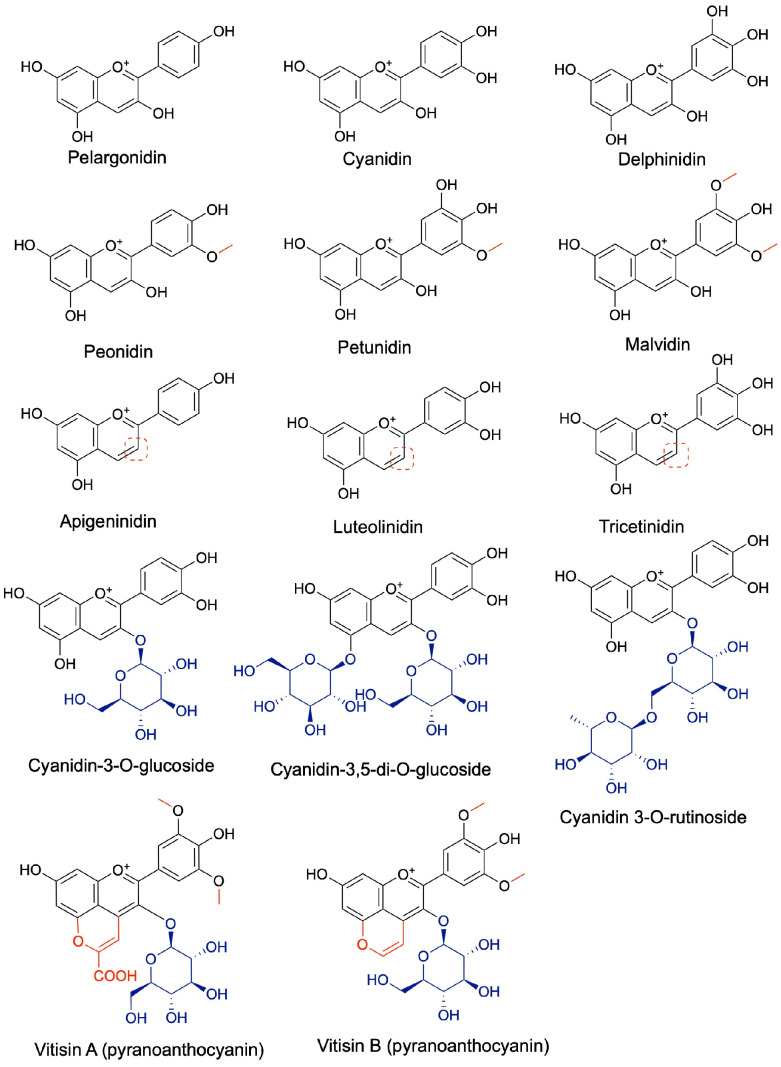
Representative structural diversity of anthocyanins and their derivatives. The anthocyanidin backbone, 3-deoxyanthocyanidins, and related compounds are compared with respect to variations in hydroxylation and methylation. Anthocyanidins (pelargonidin, cyanidin, delphinidin, peonidin, petunidin, and malvidin) are compared with their 3-deoxy analogs (apigeninidin, luteolinidin, and tricetinidin), with a dashed circle indicating the loss of the C3 hydroxyl group. Also shown are commonly occurring glycosylated cyanidins, including cyanidin-3-O-glucoside, cyanidin-3,5-di-O-glucoside, and cyanidin-3-O-rutinoside. Commonly occurring cyanidin glycosides are illustrated, including the most common cyanidin-3-O-glucoside, a less common cyanidin-3,5-di-O-glucoside, and cyanidin-3-O-rutinoside, a widely distributed disaccharide derivative. Pyranoanthocyanin (vitisin) derivatives (vitisin A and vitisin B) are also shown, which include an additional red pyran ring that increases the rigidity and stability of these molecules.

There are six naturally occurring anthocyanidin backbones: cyanidin, delphinidin, pelargonidin, petunidin, malvidin, and peonidin (Figure 2). The variations occur primarily in the number and position of the hydroxyl (–OH) and methoxy (–OCH_3_) groups in the B ring. The B-ring hydroxylations in anthocyanidins significantly alter the bioactivity by modulating the distribution of electrons, polarity of the molecule, and redox behavior. On the other hand, an increase in hydrophilicity enhances the electron-donating activity and free radical scavenging activity [31,32]. However, at the same time, the risk of oxidation and the decrease in stability of molecules with more OH groups increase [7,33]. Thus, the anthocyanidin backbone provides an optimal balance between reactivity and stability and serves as a good starting point for further modifications. The degree of B-ring hydroxylation directly modulates chromophore reactivity: a higher number of hydroxyl groups increases the nucleophilicity of the ring system, rendering the flavylium cation more susceptible to water attack at C-2 and to oxidative degradation [7,31]. These core structures are widely distributed in nature, especially in berries, grapes, and colored cereals.

### 2.2. Methoxylation

Methoxylation is the substitution of a hydroxyl group by a methoxy group (-OCH_3_), i.e., methoxylation gives peonidin (C3′-OCH_3_-cyanidin), petunidin (stepwise methoxylation of delphinidin via C3′-OCH_3_ to C3′, C3′, and C5′-OCH_3_), and malvidin (Figure 2) [31]. Methoxyl groups are electron-donating substituents that can modify the conjugated systems of molecules, thereby influencing the maximum absorption wavelength and the color of anthocyanins [34]. The methylation of hydroxyl groups to methoxyl groups reduces the number of positions susceptible to oxidation, notably on the B-ring, resulting in more stable anthocyanins that are less affected by oxidative degradation and pH changes [5]. Further, methoxylation also reduces the polarity of anthocyanins, which may influence their absorption and metabolism in vivo [35]. Moreover, some highly methoxylated anthocyanidins, such as malvidin and its derivatives, have been reported to exhibit greater stability during storage and under various conditions [36]. By reducing the number of oxidation-sensitive hydroxyl positions on the B-ring, methoxylation directly lowers the rate of oxidative chromophore degradation, contributing to the observed stability advantages of malvidin-type anthocyanins in both acidic and moderately heated food systems [5,36].

### 2.3. 3-Deoxyanthocyanidins

3-Deoxyanthocyanidins are distinguished by the absence of a hydroxyl group at the C3 position. These compounds are relatively rare in nature and have been identified mainly in sorghum, certain mosses, and ferns. Typical naturally occurring 3-deoxyanthocyanidins are apigeninidin, luteolinidin, tricetinidin, carajurin, and carajurone [29,30,37]. In common anthocyanins, changes in molecular structure under mildly acidic to neutral conditions occur mainly as hydration reactions, resulting in colorless hemiketals and yellowish chalcones [7]. The absence of a C3 hydroxyl group in 3-deoxyanthocyanidins fundamentally changes the reversible interconversion of the characteristic molecular structures of these compounds and significantly suppresses hydration reactions [29,30]. As a result, 3-deoxyanthocyanidins show significantly better color stability at high pH and maintain a visible color at near-neutral pH. This distinctive behavior arises from the unique molecular structure that gives them clear advantages over conventional anthocyanins.

### 2.4. Glycosylation

Glycosylation is the most common structural modification in naturally occurring anthocyanins. The majority of them occur as glycosides (anthocyanins) rather than in the free aglycone form (anthocyanidins) [31,32,38]. Glycosyl units occur mainly as O-glycosides at the C3 position (Figure 2), though glycosylation at C5, C7, and even the B-ring has also been documented [32,38]. Based on sugar composition, glycosylated anthocyanins are divided into monosaccharide, disaccharide, and complex glycoside forms. Most of the monosaccharides identified in glycosylated anthocyanins are glucose and galactose, but also arabinose, rhamnose, and xylose were found. The corresponding disaccharides, such as rutinosides, sophorosides, and sambubiosides, are widely reported. The role of glycosylation in determining the physicochemical properties of anthocyanins has been widely studied. Glycosylation enhances the hydrophilicity of aglycones, thereby facilitating the formation of water-soluble pigments in plants [31]. The sugar moieties are also believed to modulate the molecular conformation of the aglycone and can mediate intermolecular interactions through steric effects [39]. Recent research has clarified how glycosylation influences stability and bioavailability, showing that it can reduce hydration and conformational changes, and alter gut metabolism via interactions with the gut microbiota [38,40]. In particular, glycosylation at C3 reduces the rate of conversion to the colorless pseudobase and chalcone forms under moderately acidic to neutral conditions [7,39]. Mechanistically, the sugar moiety at C-3 creates steric hindrance around the electrophilic C-2 position of the flavylium cation, directly reducing the rate of nucleophilic water addition and slowing conversion to colorless carbinol pseudobase; the primary pathway of color loss at moderately acidic to neutral pH [39,41].

### 2.5. Pyranoanthocyanins

Pyranoanthocyanins are the derivatives formed when anthocyanins react with small molecules such as pyruvic acid, acetaldehyde, and hydroxycinnamic acids, generating a new pyran ring between the ring C4 and C5 positions. Although pyranoanthocyanins are extensively studied in wine, where they are formed during fermentation and aging, they also occur naturally in a wide range of plant tissues, including strawberries, black carrots, red onions, and various berries [42,43]. Typical pyranoanthocyanins include vitisin A, vitisin B (Figure 2), pinotin A, and oxovitisin A. Vitisins are mainly formed by the reaction of malvidin and related anthocyanidins with pyruvic acid or acetaldehyde [42,43]. Compared with common anthocyanins, pyranoanthocyanins have an additional pyran ring incorporated into the structure, resulting in a longer conjugated π-system and increased stability against pH changes, sulfite bleaching, and oxidation. Red wines contain both common anthocyanins and pyranoanthocyanins, and studies on aged wines show that the pyranoanthocyanins are the major chromophores responsible for stable color stability across a wide pH range. The additional pyran ring extends the conjugated π-electron system, reducing the electrophilicity of the chromophore and decreasing its susceptibility to nucleophilic and oxidative attack, the mechanistic basis for the superior pH and oxidative stability of pyranoanthocyanins relative to their monomeric precursors [44,45].

### 2.6. Acylation and Polyacylation

**Figure 3:**
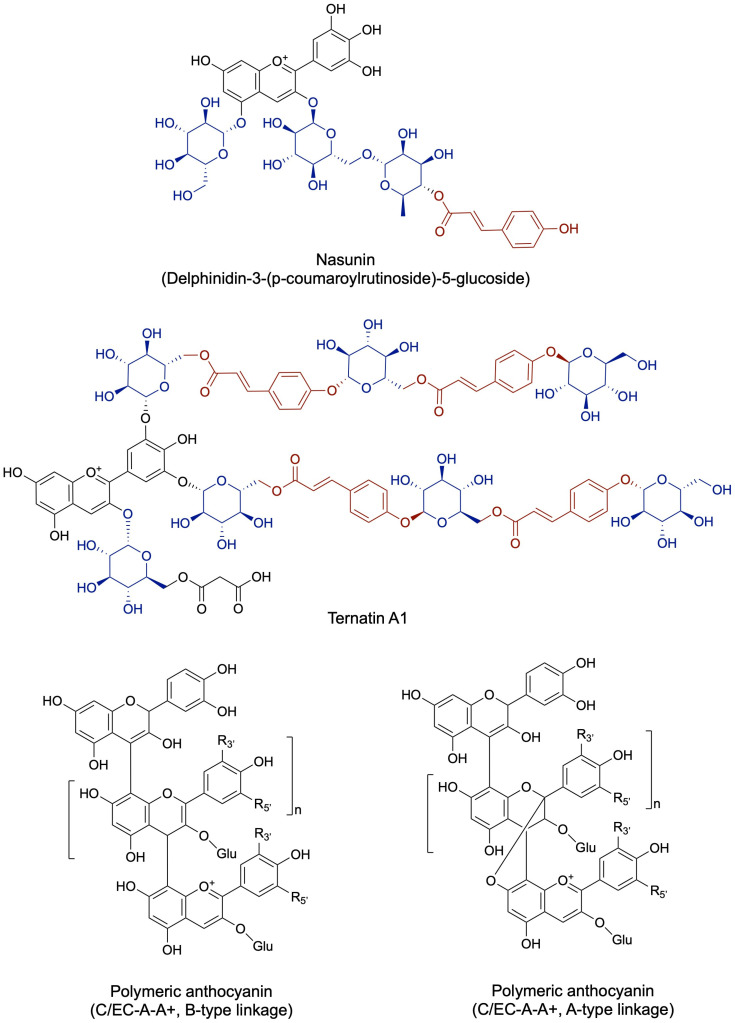
Representative structures of complex anthocyanin derivatives, showing acylation, polyacylation, and polymerization. Structures include nasunin, a mono-acylated anthocyanin (delphinidin-3-(p-coumaroylrutinoside)-5-glucoside), and ternatin A1, a highly poly-acylated anthocyanin with several aromatic acyl groups that enable intramolecular co-pigmentation. Representative polymeric anthocyanins are also shown, which form via condensation between anthocyanins and flavanols, generating B-type (single C-C bond) and A-type (additional C-O-C bond) linkages. Increased structural complexity lowers susceptibility to hydration and enhances oxidative stability and color retention.

Acylation is the glycosyl moiety of anthocyanins by esterification with organic acids such as hydroxycinnamic acids (p-coumaric acid, caffeic acid, ferulic acid, etc.) and aliphatic acids (acetic acid, malonic acid, etc.) and is commonly found in grapes and grape products [28,35]. Anthocyanins can be categorized as monoacylated and polyacylated forms according to the number and type of acyl groups [28]. Most of the anthocyanins exist in either monoacylated or polyacylated forms. Typical monoacylated anthocyanins include nasunin and delphinidin-3-(p-coumaroylrutinoside)-5-glucoside (Figure 3) from eggplant, malonylated malvidin derivatives from grapes, and acylated cyanidin derivatives from gentian. Polyacylated anthocyanins contain more than two acyl groups, either aromatic or aliphatic, exemplified by the ternatins (Figure 3) from butterfly pea and gentiodelphin-type anthocyanins (Figure 3) of gentian species [46,47]. These highly acylated pigment structures are known for their exceptional pigment stability and are widespread in nature, and can be found in a range of different plants and edible seeds. Purple sweet potato, red cabbage, and butterfly pea contain high levels of these pigments.

Acylation converts anthocyanins into acylglucosides with higher chemical stability. In these structures, the aromatic acyl units interact with the anthocyanin chromophore through π-π stacking, enhancing planarization of the conjugated ring system and thereby improving stability to hydration and oxidative damage. The bulky acyl groups also provide a physical barrier around the reactive core, shielding it from nucleophiles (e.g., water) and other environmental stresses [28,35,39,48]. In general, aromatic acylation tends to provide greater stability to anthocyanins than aliphatic acylation, and polyacylated anthocyanins are more stable than monoacylated forms. Aromatic acylation is mechanistically superior to aliphatic acylation because aromatic acyl groups possess a π-electron system capable of engaging in intramolecular π–π stacking with the flavylium chromophore, providing both electronic shielding and steric protection. Aliphatic acyl groups lack this aromatic system and contribute only steric hindrance, explaining their comparatively weaker stabilizing effect [28,49]. In acylated anthocyanins, aromatic acyl groups (e.g., p-coumaric, caffeic, or ferulic acid residues) esterified to the sugar moiety are geometrically positioned to fold back over the chromophore through intramolecular π–π stacking. This intramolecular sandwich conformation has been confirmed by NMR and computational studies, demonstrating that the acyl group adopts a folded geometry that places its aromatic ring in van der Waals contact with the flavylium chromophore. This conformation provides two independent stabilizing effects: (i) electronic shielding of the chromophore through charge–transfer interactions that reduce the electrophilicity of C-2, and (ii) steric protection, wherein the bulky acyl group physically blocks water molecules and other nucleophiles from approaching the reactive positions (particularly C-2) [49,50,51]. However, the magnitude of this stabilizing effect is not uniform across all acylated anthocyanins; it depends strongly on the number and type of acyl groups, the specific folding conformation adopted by the molecule, and the surrounding matrix conditions, which together determine the effectiveness of intramolecular stacking [49,50,51].

### 2.7. Polymeric Anthocyanins

Polymeric anthocyanin-derived pigments (Figure 3) are mainly formed through condensation reactions of anthocyanins with other phenolic compounds (e.g., flavanols, catechin, epicatechin, and other similar molecules and tannins); sometimes intermediate products (e.g., acetaldehyde) are employed as bridging molecules to form new high molecular weight pigments with complex molecular structures [32,36]. Polymeric anthocyanins commonly accumulate in wines during aging. They are formed through reactions between anthocyanins and flavanols, yielding a diverse array of higher-molecular-weight pigments that are typically darker and more stable than the original monomeric anthocyanins. Many of these polymers contain ethyl bridges between their subunits [32,36]. Polymeric anthocyanins are more stable than the monomeric anthocyanins due to increased rigidity of the polymer chain and reduced susceptibility to degradation processes, especially oxidation. These polymers are important for long-term color stability in natural systems and are among the anthocyanin-derived pigments that become stabilized through polymerization.

## 3. Structure–Property Relationships

The individual structural features described above do not act in isolation; together they define a continuum of physicochemical behavior in which each modification makes a characteristic and often predictable contribution to chromophore stability. Establishing these structure–property correlations provides a unifying framework for rationalizing the stability differences among anthocyanins and for guiding the selection of suitable structures for specific applications. The principal relationships are summarized in Table 1.

Modifying the degree and positions of hydroxylation alters the polarity and reactivity, generally increasing electron-donating activity and decreasing oxidative stability. Methoxylation, however, reduces reactivity and increases stability by decreasing the number of oxidation-sensitive sites. Glycosylation improves the water-soluble properties and generates steric barriers that inhibit hydration reactions, resulting in stability in the structure. Acylation, especially polyacylation, provides additional stability through intramolecular co-pigmentation and increased rigidity of the molecule; generally, aromatic acyl residues provide better stabilization compared to aliphatic groups.

3-Deoxyanthocyanidins are less reactive than parent anthocyanidins due to the absence of the C3 hydroxyl group, and are unexpectedly stable in near-neutral solutions. Derived structures like pyranoanthocyanins and polymeric anthocyanins are also more resistant to pH changes, oxidation, and bleaching, because of their extended conjugated systems and greater structural complexity.

## 4. Molecular Mechanism of Stability

The instability of anthocyanins under different environmental conditions originates from several distinct chemical mechanisms, including pH-dependent transformation, thermal degradation, photochemical oxidation, and copigmentation-mediated stabilization. Understanding these mechanisms provides the chemical foundation for the structure-dependent stability differences discussed in subsequent sections.

### 4.1. pH-Dependent Structural Transformation

The pH-dependent color loss of anthocyanins proceeds through a well-defined sequence of chemical transformations. Under strongly acidic conditions, the flavylium cation (AH^+^) is thermodynamically stable and confers intense red coloration. As pH increases toward mildly acidic and neutral conditions, two competing reactions occur simultaneously: (i) proton transfer from AH^+^ yields the neutral quinoidal base (A) and its anionic form (A^−^), responsible for blue-purple hues; and (ii) nucleophilic addition of water at the electrophilic C-2 position generates the colorless carbinol pseudobase (B). The carbinol form subsequently undergoes ring-opening tautomerization to yield the pale yellow cis-chalcone (Cc), which slowly isomerizes to the more stable trans-chalcone (Ct). At near-neutral to alkaline pH, the equilibrium strongly favors the colorless B/Cc/Ct forms, resulting in effective color loss [9,25,26]. These sequential transformations correspond to the reversible equilibria illustrated in Figure 1**.** It is important to note that color changes in anthocyanins reflect underlying structural transformations of the chromophore, which can also affect bioactivity. The flavylium cation form, dominant at low pH, is generally associated with stronger antioxidant and free-radical scavenging activity than the colorless hemiketal and chalcone forms generated at higher pH. Therefore, conditions that destabilize the colored form may simultaneously diminish the health-related functionality of anthocyanins [31].

### 4.2. Thermal Degradation Pathway

Thermal degradation of anthocyanins proceeds primarily through two pathways: (i) hydrolysis of the glycosidic bond at elevated temperatures, releasing the less stable aglycone; and (ii) cleavage of the C-ring, particularly opening of the pyran ring, yielding degraded chromophores with reduced color intensity. The activation energy for these reactions is strongly influenced by molecular rigidity; structures with intramolecular π–π stacking (e.g., polyacylated anthocyanins) present a higher kinetic barrier to thermal unfolding and chromophore exposure, thereby retarding degradation [33,49,50].

### 4.3. Photochemical and Oxidative Pathway

Photodegradation of anthocyanins is primarily initiated by singlet oxygen (^1^O_2_) and reactive oxygen species (ROS) generated upon light absorption, which attack the electron-rich positions of the chromophore. Structures with reduced hydroxyl group density (methoxylation) or with shielded chromophores (acylation) present fewer accessible oxidation sites, thereby retarding this pathway [52,53].

### 4.4. Copigmentation and Acylation-Mediated Stabilization

**Figure 4:**
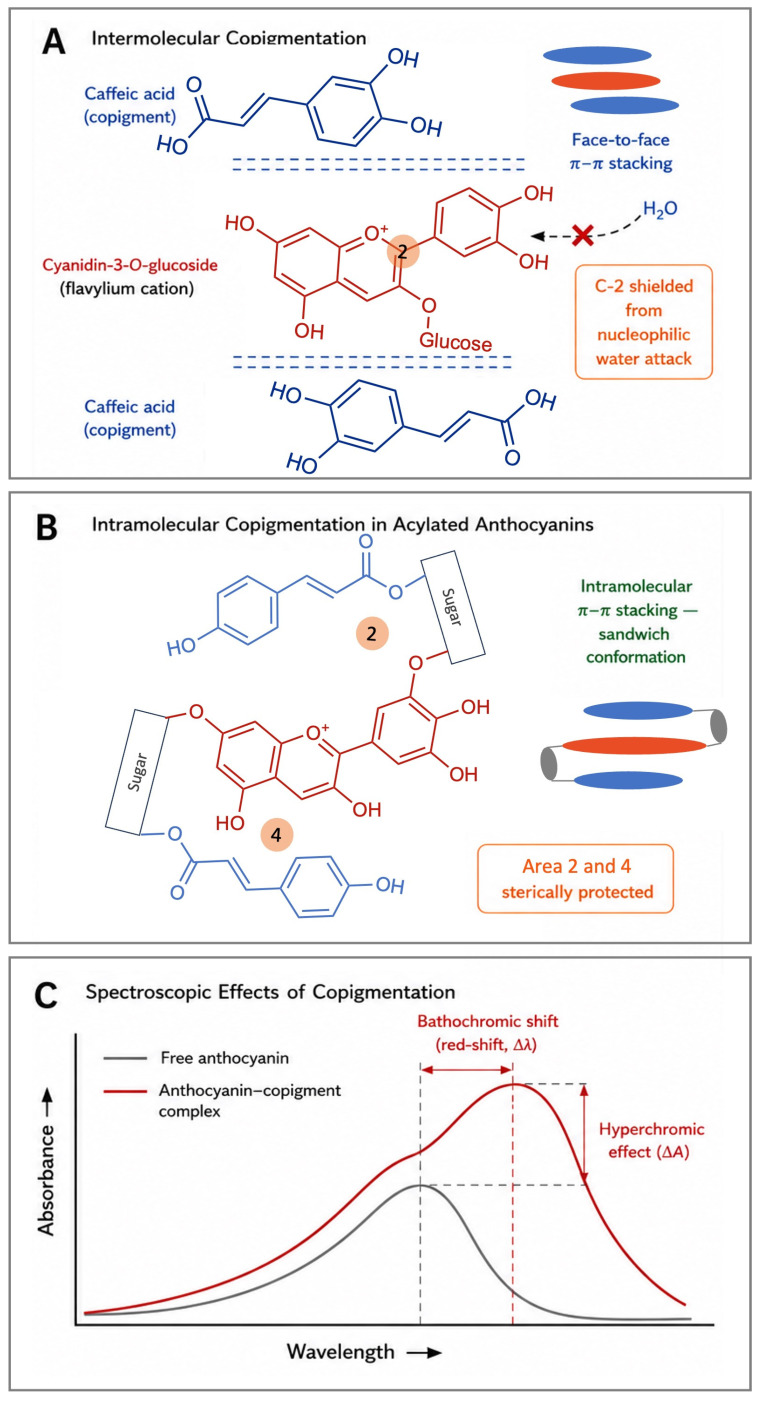
Molecular mechanisms of copigmentation and acylation-mediated stabilization of anthocyanins. (**A**) Intermolecular copigmentation: An electron-rich aromatic copigment (e.g., caffeic acid) interacts with the electron-deficient flavylium chromophore through face-to-face π–π stacking, physically shielding the electrophilic C-2 position from nucleophilic water attack. (**B**) Intramolecular copigmentation in acylated anthocyanins: Aromatic acyl groups attached to the sugar moiety fold back over the chromophore, forming a “sandwich” conformation that provides both electronic shielding and steric protection at the reactive positions. (**C**) Characteristic UV–visible spectroscopic effects of copigmentation: the copigmented complex exhibits a bathochromic shift (red-shift of λmax) and a hyperchromic effect (increased absorbance) relative to the free anthocyanin, reflecting the perturbation of the chromophore electronic environment.

At the molecular level, copigmentation is governed by π–π stacking interactions between the electron-deficient flavylium chromophore and the aromatic ring systems of electron-rich copigments such as phenolic acids, flavonoids, and alkaloids. This stacking geometry positions the copigment in a face-to-face or offset arrangement above and/or below the chromophore plane. The resulting hydrophobic stacking complex produces two characteristic spectroscopic effects: a bathochromic shift (red-shift of λmax) and a hyperchromic effect (increase in absorbance intensity), as shown in Figure 4. More importantly, from a stability perspective, the stacked copigment physically shields the electrophilic C-2 position of the flavylium cation from nucleophilic attack by water, thereby suppressing the hydration equilibrium and stabilizing the colored form at pH values where it would otherwise be lost [9,17,50,54,55,56].

## 5. Intrinsic vs. Extrinsic Stability Factors

Anthocyanin stability is governed by both intrinsic molecular features and extrinsic environmental factors, which are examined separately below.

### 5.1. Intrinsic Molecular Factors

A growing body of research shows that molecular structure is key to how anthocyanins respond to environmental stress. For this reason, clarifying these structure-related stability differences is crucial for understanding the behavior of anthocyanins in food and biological systems, as outlined in Table 2.

Substitution patterns on the B-ring were found to play a very important role. A higher degree of hydroxylation on the B-ring increases nucleophilic susceptibility, promoting hydration and oxidative degradation. Methoxylation of the B-ring reduces the number of reactive sites and imparts chromophore stability, possibly due to its electron-donating effect [46,50]. Glycosylation, particularly glycosylation at the C3 position, causes sterical hindrance, which makes the electrophilic C2 position less susceptible to reaction with water, thus reducing the rate of hydration [41]. Acylation seems to be the best method of molecular stabilization of chromophores. Co-pigmentation with intramolecular folding of the acyl group over the chromophore leads to significant stiffening of the molecule, thus preventing hydration reactions at slightly acidic to nearly neutral pH of the solution [49,50]. In contrast, 3-deoxyanthocyanidins are less susceptible to hydration because they lack the C3 hydroxyl group, the key site for carbinol pseudobase formation; such a structural trait allows them to retain good color stability at higher pH values [29,30,50].

Acylation is the dominant stabilizing factor, with polyacylated anthocyanins exhibiting enhanced thermal resistance due to intramolecular stacking and steric shielding of the chromophore [33,49,50]. Methoxylation further improves stability, whereas highly hydroxylated structures are more prone to degradation [33,50]. Glycosylation contributes to general stability but plays a secondary role under thermal stress [33,53]. In addition, 3-deoxyanthocyanidins may exhibit improved thermal stability due to reduced susceptibility to hydration and structural transformation [37,57]. Polymeric anthocyanin-derived pigments also tend to show enhanced thermal stability, which can be attributed to increased molecular rigidity and structural complexity [58,59].

The sensitivity of anthocyanins to light and oxidation is influenced by both the molecular electronic structure and the availability of oxidation sites within the molecule. Anthocyanins with multiple hydroxyl groups are more likely to undergo oxidative degradation, as these groups increase electron transfer reactivity. However, methoxylation reduces these reactive positions, thereby increasing the resistance to degradation [52,53].In addition, the acylation effect stabilizes the chromophore by preventing intermediate states and their reactivity and by enhancing the intramolecular interactions [49].

Some derived structures, such as pyranoanthocyanins and polymeric anthocyanins, can be more stable than parent anthocyanins due to increased conjugation and enhanced rigidity of the resulting molecules, thereby making them less susceptible to photodegradation and oxidative bleaching [44,45,53]. Similarly, 3-Deoxyanthocyanidins may be more stable to oxidative degradation than conventional anthocyanins due to their stable electronic structures with fewer reactive hydroxyl groups [29,30].

Pyranoanthocyanins and polymeric anthocyanins are more stable in food environment due to increased rigidity of macromolecule chains and decreased sensitivity to external factors [58,59,60,61]. Redox-active additives, such as ascorbic acid, that catalyze oxidative reactions leading to the degradation of the flavylium cation can cause some degree of chromophore breakdown; however, acylation and methoxylation of the ring system offer some stabilization by making the flavylium cation less reactive to oxidation [41,49,60].

### 5.2. Extrinsic Molecular Factors

Anthocyanin stability is strongly affected by environmental conditions, including pH, temperature, light, and oxygen, all of which can trigger structural changes and chemical degradation.

Changes in color and instability of anthocyanins at different pH values are mainly due to the shift in chemical equilibrium of the flavylium cation (AH+), the quinoidal base (A), and the hydrated structures (B/Cc/Ct) (Figure 1) [27,52,62]. The pH range over which these transitions occur varies considerably depending on molecular structure. For cyanidin-3-glucoside, a representative common anthocyanin, the flavylium cation (AH^+^, λmax ≈ 512 nm) dominates below pH 2; the apparent pKa for the deprotonation equilibrium is approximately 3.8 in aqueous solution, indicating that hydration to the colorless hemiketal becomes thermodynamically favorable as pH approaches 4. A local minimum in molar absorptivity is observed near pH 4, and the equilibrium is essentially fully shifted to the hemiketal, quinoidal base, and chalcone forms at pH 5–7 [9,26]. Aromatic acylation substantially elevates the effective pKh by introducing intramolecular π–π stacking that shields C-2, thereby extending meaningful color retention toward near-neutral pH [49,50]. The 3-deoxyanthocyanidins (e.g., luteolinidin, apigeninidin) represent a structurally distinct class: the absence of the C3 hydroxyl group fundamentally suppresses the hydration pathway, enabling visible color retention across a markedly broader pH range than is observed for common anthocyanins [30,37,57]. It should be noted that precise pH transition thresholds are compound-specific and cannot be generalized to fixed values across all anthocyanin structures. Overall, pH stability is associated with resistance to hydration and preservation of the flavylium chromophore.

Thermal stability is primarily determined by structural features that enhance molecular rigidity and reduce susceptibility to chemical degradation. Overall, thermally stable anthocyanins tend to have fewer reactive sites and greater structural rigidity.

The stability of anthocyanin in complex systems depends on the compatibility of its structure with other components. Interactions of anthocyanins with proteins and polysaccharides are also affected by their chemical modifications, such as glycosylation and acylation, which change their polarity. The complexes formed by these interactions can stabilize anthocyanins but can also cause unwanted self-aggregation and color degradation. These phenomena are studied in protein–anthocyanin and polysaccharide interaction systems [41,54,63]. Co-pigmentation and metal ion complexation interactions, which are effective for improving color stability, also require structural features, particularly the substituents on the B-ring [34,54,63].

Pyranoanthocyanins and polymeric anthocyanins are more stable in a food environment due to increased rigidity of macromolecule chains and decreased sensitivity to external factors [58,59,60,61]. Redox-active additives, such as ascorbic acid, that catalyze oxidative reactions leading to the degradation of the flavylium cation can cause some degree of chromophore breakdown; however, acylation and methoxylation of the ring system offer some stabilization by making the flavylium cation less reactive to oxidation [41,49,60]. These matrix-mediated effects form the practical basis for the structure-dependent application strategies discussed in the following section [54,64].

## 6. Implications for the Food System

**Figure 5:**
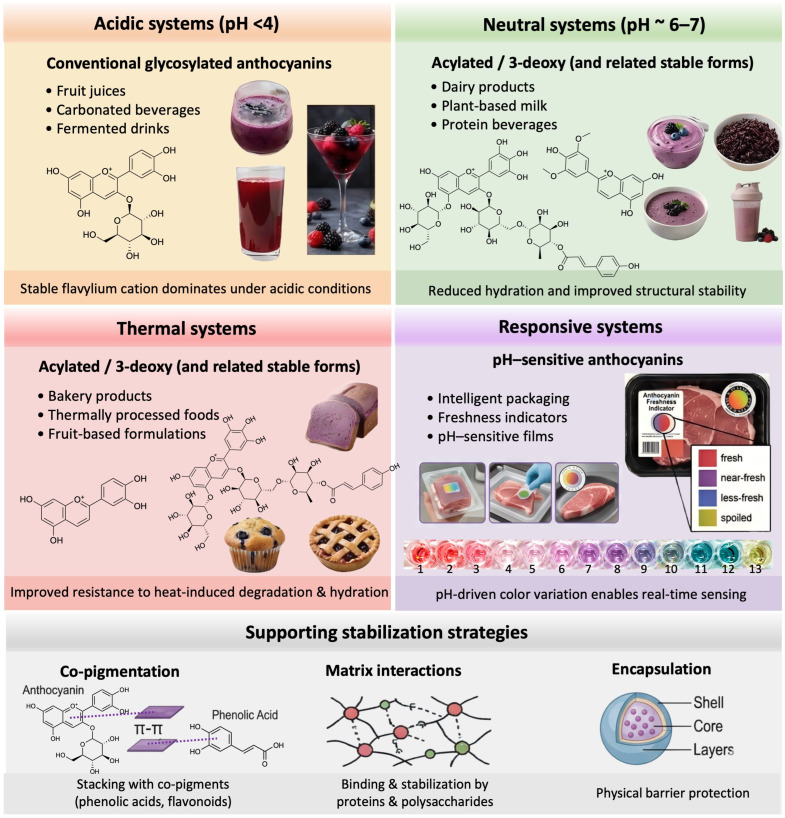
Structure-based suitability of anthocyanins for different food systems and application scenarios. The behavior of anthocyanins in food matrices is primarily influenced by the interaction between their molecular structure and the surrounding environment. At a pH below 4, conventional glycosylated anthocyanins function optimally owing to the dominance of the flavylium cation. Conversely, at a pH of around 6–7, anthocyanins exhibiting structural modifications, such as acylation or the absence of a C3 hydroxyl group (3–deoxyanthocyanidins), are more advantageous, as they exhibit reduced susceptibility to hydration and enhanced structural stability. In thermal processing systems, these altered anthocyanins exhibit enhanced resistance to degradation and hydration processes. pH–sensitive color-changing anthocyanins are especially beneficial for responsive systems such as intelligent packaging and freshness indications. Stabilization processes, such as co-pigmentation, interactions with proteins and polysaccharides in the food matrix, and encapsulation, can augment anthocyanin stability and broaden their practical applications.

The application of anthocyanins in food systems depends not only on their color properties but also on the environmental constraints of food systems. From a practical point of view, the stability of anthocyanins must be related to the main processing and storage factors that affect their potential utilization in food products and systems. Variations in structure, such as differences in substitution patterns or chemical modifications, influence the color stability of anthocyanins in different ways, depending on their use. In food applications, a range of conditions are encountered, from acidic or neutral environments to high-temperature processing for human consumption. Typically, additional stabilization is needed to support the inherent instability of anthocyanins. The structure-based suitability for use in various food systems and for specific purposes is shown in Figure 5. Food processing by-products and waste streams represent increasingly important sources of anthocyanins. Berry pomace, grape skins and seeds from winemaking, red cabbage processing residues, and purple sweet potato peels are rich in anthocyanins and are gaining attention for sustainable colorant production within circular bioeconomy approaches [65,66].

### 6.1. Acidic Systems

The stability of anthocyanins in acidic food systems like fruit juices, carbonated drinks, and fermented drinks depends considerably on maintaining the flavylium cation. Flavylium cations are what give them their red hue. The equilibrium moves toward the flavylium cation when the pH is low, which is the most common type. The acidic environment stabilizes this cation, therefore the structural needs for anthocyanins are not as strict [22,67,68].

Structural differences between anthocyanins can still affect their secondary degradation pathways, especially when exposed to heat, light, or oxygen. Conversely, conventional glycosylated anthocyanins typically prove adequate within these acidic systems, provided the acidic environment is preserved. In practical contexts, anthocyanins derived from berries find extensive application in beverages and juice products, owing to their favorable performance under low pH conditions. Commercial applications include the use of black carrot anthocyanins in fruit juices and carbonated beverages, grape skin extracts in soft drinks, and elderberry concentrates in functional beverages [65,66,68,69]. In fermented products such as wine, free anthocyanin content typically peaks during the early extraction phase, when anthocyanins are released from plant tissues into the must. During subsequent fermentation and aging, the content of free monomeric anthocyanins gradually declines as they are progressively converted through several parallel reactions: condensation with flavanols and tannins to form polymeric pigments, reaction with small molecules such as pyruvic acid and acetaldehyde to form pyranoanthocyanins, and partial oxidative degradation [61,70]. These three pigment classes differ markedly in their long-term stability: monomeric anthocyanins are the most susceptible to hydration and degradation; pyranoanthocyanins, owing to their additional pyran ring and extended conjugation, are substantially more resistant to pH change and bleaching; and polymeric pigments, formed through condensation with flavanols and tannins, provide the most durable color contribution and become the dominant chromophores in aged wines [61,70].

The color characteristics of anthocyanins in acidic food systems are predominantly influenced by environmental conditions, rather than structural optimization. As a result, establishing a suitable pH and controlling the environment are more important than modifying the molecular structure of anthocyanins.

### 6.2. Neutral Systems

Anthocyanins are difficult to apply in near-neutral systems such as dairy products, plant-based milk substitutes, and protein-enriched beverages. The flavylium cation is less unstable at such pH levels and readily hydrates into colorless forms. As a result, anthocyanin-containing products in near-neutral systems often undergo rapid quality deterioration, including changes in perceived color and reduced acceptability [67]. To cope with this issue, the stability of anthocyanins can be further enhanced by restricting hydration and conferring rigidity on the molecules. The acyl groups of anthocyanins achieve this effect by “fixing” the molecules into a rigid structure, thereby enhancing stability via intramolecular co-pigmentation [49,51,71]. Additionally, methoxyl groups generally improve the stability of anthocyanins. The 3-deoxy analogs shown here are also stable, exhibit limited reactivity towards hydration, and remain deep red under near-neutral conditions [29,30,51].

The inherent structural stability of anthocyanins is crucial in near-neutral conditions; additionally, their interactions with other food matrix components might further enhance their stability. Protein–anthocyanin and polysaccharide–anthocyanin binding are two examples of these kinds of interactions that have been studied in dairy-like model systems with whey proteins or casein [64,72]. These matrix effects are further strengthened by co-pigments like phenolic chemicals, which enhance non-covalent bonds and promote better stability during storage. These interactions not only preserve color but also enhance solubility and bioavailability, thereby expanding the functional applications of anthocyanins in intricate food systems [8,73,74]. Despite these strategies, the inherent instability of anthocyanins at neutral pH still restricts their use unless structural stabilization or matrix-assisted methods are used. Therefore, neutral systems represent the most structure-sensitive application scenario, where intrinsic molecular properties and matrix interactions must be considered together. In neutral systems, anthocyanins from butterfly pea flower (predominantly polyacylated ternatins) are used in plant-based beverages and dairy alternatives [46,47].

### 6.3. Thermal Systems

Thermally processed systems, including baked goods, pasteurized beverages, and heat-treated sauces, can significantly undermine the stability of anthocyanins by accelerating degradation reactions as temperatures rise [68,75]. Under these conditions, structural features that enhance molecular rigidity and reduce exposure to reactive sites are particularly important. Acylated anthocyanins are particularly advantageous for these applications due to intramolecular co-pigmentation, which stabilizes the chromophore and reduces thermal degradation [67,76]. This structural advantage has been consistently recorded in naturally acylated anthocyanin sources. For instance, black carrot anthocyanins that are highly acylated are more stable at high temperatures and can be used for processing at high temperatures [67,68,77].

In practical applications, structurally stabilized anthocyanins are widely used in thermally processed food systems, including bakery fillings and jams, confectionery (e.g., gummies and hard candies), fruit-based formulations, and ice cream, where preserving color intensity throughout processing is important [78,79].

### 6.4. Responsive Systems

Despite the instability of anthocyanins, which limits their use in many fields, they hold significant value in specific applications, particularly intelligent packaging and freshness indicator systems. Here, their intrinsic instability becomes a functional advantage rather than a limitation. This is because their color changes in response to environmental fluctuations, especially pH shifts, allowing for real-time monitoring of food quality, such as tracking freshness through reversible color variations [80,81,82,83]. In these systems, structural responsiveness is more important than structural stability. Anthocyanins that may undergo predictable and discernible color changes are desirable, as their transformation characteristics directly influence sensing performance. The reversible transformation between flavylium and quinoidal species in response to varying pH levels enables visual illustration of spoilage-related changes [84,85,86,87]. This behavior has been extensively employed in pH-sensitive indicator films to monitor the freshness of meat and seafood products. The color changes in these films are correlated with biochemical deterioration processes. In response to pH shifts caused by protein degradation in products such as crustaceans or meat, anthocyanin-incorporated films can display distinct color transitions (e.g., red to blue or green) [84,85,86,87]. Biopolymers such as chitosan and other polysaccharides are commonly used in the construction of these systems, thereby enabling interaction with volatile compounds or pH fluctuations while preserving a controlled microenvironment.

A major challenge in these applications is achieving an equilibrium between stability and responsiveness. Structures with excessive stabilization can experience lowered sensitivity, whereas excessive degradation could undermine the reliability of the signal. Therefore, to achieve the best possible balance between stability and responsiveness, the effective design of responsive systems requires careful consideration of structure-dependent transformation behavior. Such anthocyanin-based intelligent packaging systems are increasingly being explored for commercial deployment in cold-chain monitoring of fresh and processed foods [88,89].

### 6.5. Stabilization Strategies in Support of Application

Although the structural characteristics of anthocyanins determine their basic stability, additional measures are often needed for use in adverse conditions where their stability is inadequate. At the molecular level, the stability of anthocyanins can be enhanced through co-pigmentation with co-pigments like simple phenolic acids or flavonoids via non-covalent interactions, which can stabilize the chromophore’s electronic structure, making it less prone to hydration and degradation, thus improving color intensity and stability.

The interaction with macromolecules such as proteins and polysaccharides can provide stability from a matrix point of view. The interaction with macromolecules increases stability by providing a different local microenvironment and decreases the exposure of anthocyanin to reactive agents. Such an interaction has been shown in systems with whey protein and pectin [72,73]. On a structural basis, physical approaches such as microencapsulation and other structured systems provide physical barrier environments that protect against external influences like oxygen, light, and moisture. Through this process, stability is improved, enabling its application in formulated food systems [90,91,92]. However, in most cases, efficient stabilization requires the synergistic interplay between the molecular interaction, the matrix effect, and the structural protection process, and it is necessary to apply further stabilization methods to enhance the properties of conventional anthocyanins.

## 7. Conclusions and Future Perspectives

Anthocyanins are a group of natural pigments that vary in color and stability depending on their chemical structure and environmental factors. This review summarizes the instability of these remarkable compounds and discusses the structural factors that determine this instability, including the electronic effects of substituents (e.g., hydroxy or methoxy groups), steric effects (e.g., large substituents such as glycosides or acyl groups), and the intrinsically stable core frameworks, including 3-deoxyanthocyanidins and pyranoanthocyanins.

Although a good understanding of the factors influencing the stability of anthocyanins has been accumulated, translating this knowledge into food products remains challenging. Most of the research has been conducted using model systems; nevertheless, actual food systems are considerably more complicated. In addition to molecular interactions, other factors such as pH, temperature, oxygen, light, and interaction with other food components can also affect hydration, oxidation, and conformation of anthocyanins and therefore their stability in real food systems. Some anthocyanins, such as 3-deoxyanthocyanidins, acylated anthocyanins, and pyranoanthocyanins, are known to be intrinsically more stable. However, it is important to note that the behavior of anthocyanins in food systems might be quite different from that in the results obtained in simple model studies.

Future studies should connect theory and practice. This includes (1) paying more attention to naturally stable anthocyanins, such as acylated anthocyanins, pyranoanthocyanins, and 3-deoxyanthocyanidins, which demonstrate stability under harsh conditions, including variations in temperature, pH, and oxidation; (2) using real food systems other than relying solely on simple model studies, which often overlook the complexity of various processes and factors; (3) selecting appropriate stabilization techniques based on specific structural features; and (4) integrating computer modeling with experiments to better understand the anthocyanin stability in practical applications. Progress in research within these fields will facilitate the shift in anthocyanin production from empiricism to more science-based practices, which will enable the development of reliable and customized natural dyes for the food industry.

## Figures and Tables

**Table 1:** Structure–property relationships of anthocyanins.

Structural Feature	Structural Variation	Mechanistic Effect	Stability Implication	Representative Example
Hydroxylation	Variation in OH number and position (mainly on B-ring)	Increased polarity, nucleophilicity, and electron donation	Higher hydroxylation generally reduces stability and promotes hydration/oxidation	Cyanidin, delphinidin
Methoxylation	OCH_3_ substitution (primarily on B-ring)	Reduced reactivity; fewer oxidation-sensitive sites	Improved oxidative and pH stability	Malvidin, peonidin, petunidin
3-Deoxyanthocyanidins	Absence of C3 hydroxyl group	Altered structural equilibrium; suppressed hydration pathway	Reduced hydration sensitivity; color retained at higher pH	Apigeninidin, luteolinidin
Glycosylation	Sugar substitution (mainly at C3)	Increased hydrophilicity; steric shielding of C-2	Enhanced solubility and baseline stability; delayed hydration	Cyanidin-3-O-glucoside
Pyranoanthocyanins	Formation of an additional pyran ring	Extended π-conjugation; reduced chromophore electrophilicity	High resistance to pH change, oxidation, and sulfite bleaching	Vitisin A, vitisin B
Acylation	Aromatic or aliphatic acyl groups on sugar moiety	Intramolecular π–π stacking; increased rigidity	Strong stabilization (aromatic > aliphatic; poly > mono)	Nasunin, ternatin A1
Polymeric anthocyanins	Condensation with flavanols/tannins	Increased molecular complexity and rigidity	Improved long-term color stability	Anthocyanin–flavanol adducts (aged wine pigments)

**Table 2:** Structure-dependent stability of anthocyanins under environmental conditions.

Structural Feature/Modification	pH Stability	Thermal Stability	Light & Oxidative Stability	Stability in Complex Matrices	Key Mechanism
B-ring hydroxylation	↓ Promotes hydration	↓ Increased degradation	↓ Higher oxidative susceptibility	Affects co-pigmentation and metal interactions	Higher nucleophilicity and electron transfer reactivity
B-ring methoxylation	↑ Stabilizes chromophore	↑ Improves thermal resistance	↑ Enhances oxidative resistance	Reduced sensitivity to redox-active components	Reduced reactive sites and enhanced chromophore stabilization
Glycosylation (C3)	→ Delays hydration	→ Minor effect	→ Slight protection	Modulates polarity and macromolecular interactions	Steric hindrance against water attack (C2 protection)
Acylation (intramolecular co-pigmentation)	↑ Suppresses hydration	↑ Major stabilizing factor	↑ Reduces oxidative degradation	Improved matrix compatibility	Intramolecular stacking and steric shielding
3-Deoxyanthocyanidins	↑ Improved stability at higher pH	↑ Enhanced thermal stability	↑ Improved oxidative resistance	Improved persistence in complex matrices	Reduced hydration due to the absence of C3–OH
Pyranoanthocyanins	→ More stable forms	→ Improved thermal stability	↑ Strong resistance to photodegradation and oxidation	Enhanced stability in complex matrices	Extended conjugation and increased structural rigidity
Polymeric anthocyanins	→ Stable forms	→ Improved thermal stability	↑ Very high resistance to oxidative degradation	High persistence in complex systems	Polymerization and increased molecular complexity

## Data Availability

No new data were created or analyzed in this study. Data sharing is not applicable to this article.
